# The Effect of Nostril Retainer Use on Nostril Aperture Symmetry After Primary Aesthetic Rhinoplasty

**DOI:** 10.3390/medicina62081436

**Published:** 2026-07-24

**Authors:** Nagihan Gülhan Yaşar, Ağah Yeniçeri, Burak Hazır, Ayşe Seçil Kayalı Dinç, Melih Çayönü

**Affiliations:** Department of Otorhinolaryngology, Head and Neck Surgery, Ankara City Hospital, Ankara 06800, Turkey; agahyeniceri@gmail.com (A.Y.); b_hazir@hotmail.com (B.H.); kayalidincsecil@gmail.com (A.S.K.D.); melihcayonu@gmail.com (M.Ç.)

**Keywords:** rhinoplasty, nostril retainer, nostril symmetry, nasal base, ImageJ

## Abstract

*Background and Objectives*: Nostril aperture symmetry is an important component of nasal base aesthetics after rhinoplasty. However, nostril retainers are commonly used in reconstructive nasal surgery; their role after primary aesthetic rhinoplasty remains insufficiently defined. This study aimed to evaluate the effect of early postoperative nostril retainer use on nostril aperture symmetry after primary rhinoplasty using ImageJ-based morphometric measurements. *Materials and Methods*: This prospective, non-randomized comparative observational study included 52 patients who underwent primary open structural rhinoplasty. A total of 26 patients used a nostril retainer postoperatively, whereas 26 patients did not. Standardized basal-view photographs were obtained at postoperative week 1 and postoperative month 3. Right and left nostril aperture area, height, and width were measured using ImageJ software version 1.54 g after individual calibration with a millimetric ruler. Changes in symmetry indices from week 1 to month 3 were compared between groups. *Results*: Baseline week-1 symmetry indices were comparable between groups. The increase in area symmetry index from postoperative week 1 to month 3 was significantly greater in the nostril retainer group than in the control group (3.08 ± 4.99 vs. −1.00 ± 4.86; mean difference: 4.08; 95% CI: 1.34–6.82; *p* = 0.004). Changes in height symmetry index and width symmetry index did not differ significantly between groups. *Conclusions*: Early postoperative nostril retainer use was associated with improved nostril area symmetry after primary rhinoplasty. This benefit may be better reflected in global area-based symmetry than in isolated height or width measurements. Nostril retainers may represent a simple, noninvasive adjunct for selected patients at risk of early postoperative nostril asymmetry.

## 1. Introduction

Nostril shape and right–left symmetry are critical determinants of nasal base aesthetics following rhinoplasty. Despite achieving a satisfactory dorsal profile and nasal tip, asymmetry of the nostril aperture can negatively affect the perceived surgical outcome, particularly in basal-view assessments. Consequently, objective and reproducible evaluation of postoperative nasal morphology has become increasingly important [1]. Digital measurements obtained from standardized clinical photographs provide quantitative data for assessing nasal shape and symmetry [2].

Postoperative contour of the nostril depends on the surgical technique, early wound healing, scar remodeling, tissue memory and contractile forces around the alar rim and nostril aperture. During reconstructive nasal surgery for cleft-related nasal deformity and nostril stenosis, postoperative splints or nostril retainers are used to support healing tissues, minimize contraction, and maintain the corrected nostril shape [3,4].

Nostril retainers are commonly used in reconstruction procedures, but their role after primary aesthetic rhinoplasty is less clearly defined. Most existing evidence focuses on general nasofacial anthropometric parameters, nasal contour, or patient-reported outcomes [5]. However, objective data specifically assessing right–left nostril aperture symmetry after primary rhinoplasty are limited.

This study evaluated the effect of early postoperative nostril retainer use on nostril aperture symmetry after primary rhinoplasty, using calibrated basal-view photographs and ImageJ-based morphometric measurements. It was hypothesized that nostril retainer use would be associated with greater improvement in nostril area symmetry compared to no retainer use.

## 2. Materials and Methods

### 2.1. Study Design and Population

This prospective, non-randomized comparative observational study evaluated the effect of postoperative nostril retainer use on nostril aperture symmetry following primary rhinoplasty. After ethical approval, patients undergoing primary rhinoplasty between October 2024 and January 2026 were followed according to a standardized photographic protocol. Fifty-two patients who underwent primary rhinoplasty by a single surgeon at the Department of Otorhinolaryngology–Head and Neck Surgery, Ankara Bilkent City Hospital, were included. The cohort consisted of 26 patients who used a nostril retainer postoperatively and 26 patients who did not. Postoperative nostril retainer use was determined by the surgeon’s clinical judgment rather than random allocation.

Inclusion criteria were primary rhinoplasty and availability of standardized basal-view photographs at postoperative week 1 and postoperative month 3. Exclusion criteria included revision rhinoplasty, previous nasal surgery, cleft lip-palate or congenital craniofacial deformity, post-traumatic nasal deformity, severe preoperative tip deformity or alar retraction requiring alar rim grafts or other complex grafting techniques, postoperative wound-healing complications, inadequate photographic quality, or non-compliance with the retainer protocol.

### 2.2. Surgical Procedure and Nostril Retainer Protocol

All patients underwent primary structural open rhinoplasty under general anesthesia using a standardized surgical approach. Access was obtained through a Goodman-type inverted-V transcolumellar incision combined with bilateral marginal incisions. The nasal dorsum was exposed through supraperichondrial and subperiosteal dissection. Septoplasty was performed in all cases, and septal cartilage was harvested for grafting. Dorsal hump reduction was performed in a component-based manner. The cartilaginous hump was reduced with dorsal scissors, and the bony portion was reduced with an osteotome and refined when necessary. Bony vault narrowing was achieved using transverse osteotomies with a manual saw and lateral osteotomies with a lateral osteotome. Tip surgery was performed using a structural approach. Cephalic resection of the lateral crura was performed, preserving at least 8–10 mm of cartilage. Dome-binding sutures refined the tip contour. Spreader grafts were placed in all patients. Tip support was standardized with a flexible tongue-in-groove technique [6] and a columellar strut graft. Additional cap grafts were added as indicated by intraoperative findings. Alar rim grafts and alar base surgery were not performed in any patient. Skin and mucosal incisions were closed with either 5-0 rapidly absorbable polyglactin or 6-0 polypropylene sutures, and all patients received standard postoperative care.

**Figure 1:**
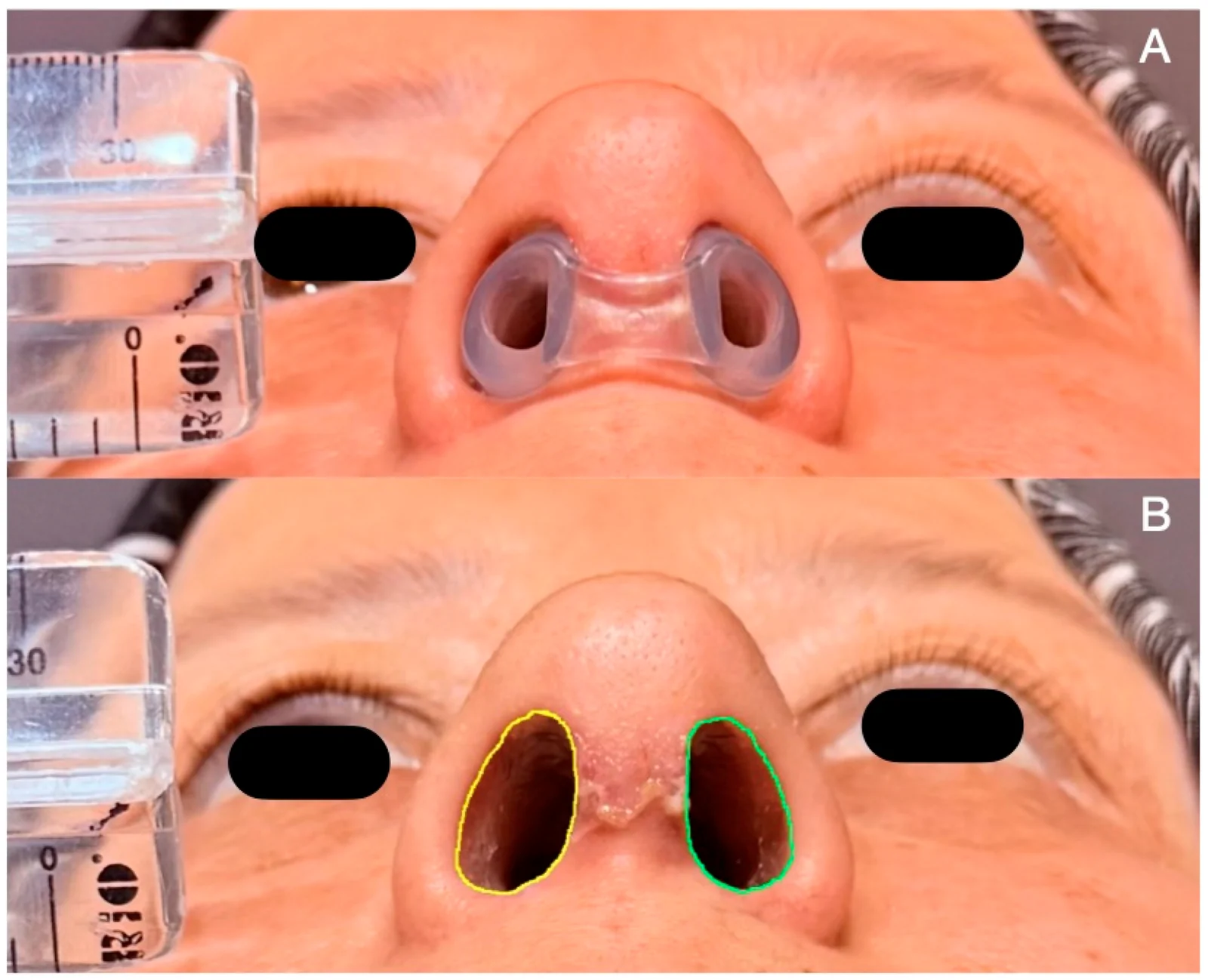
Nostril retainer placement and ImageJ-based measurement method. (**A**) Representative basal-view photograph showing bilateral nostril support with the retainer. (**B**) Manual outlining of both nostril apertures in ImageJ after ruler-based calibration for area, height, and width measurements.

Prior to retainer use, the appropriate nostril retainer size was determined individually for each patient using sizing dummies. A commercially available nostril retainer, described by the manufacturer as a silicone nostril-shaping device (Erfecen Medical, Istanbul, Türkiye), was used in all patients allocated to the retainer group. After size selection, the retainer was positioned to provide gentle bilateral support to the nostril apertures without exerting excessive pressure on the alar rim, columella, or vestibular soft tissues. Representative retainer positioning is shown in Figure 1A.

For the nostril retainer group, retainer use was initiated after the postoperative week-1 basal-view photographs. Patients were instructed to wear the retainer continuously for the first two weeks, followed by nighttime use for the subsequent two weeks. The total duration of retainer use was four weeks, with discontinuation at postoperative week 5. Retainer adherence was assessed verbally at the completion of the retainer-use period during the postoperative week-5 follow-up visit, and patients who did not comply with the prescribed retainer protocol were excluded from the study.

The final photographic assessment was performed at postoperative month 3. The control group did not use a nostril retainer.

### 2.3. Photographic Protocol and ImageJ Measurements

Standardized basal-view nasal photographs obtained at postoperative week 1 and postoperative month 3 were analyzed. All photographs were captured using the same smartphone camera system (iPhone 16 Pro Max, Apple Inc., Cupertino, CA, USA). Photographs were captured using 2× zoom at a fixed camera-to-subject distance of 1.5 m under consistent lighting conditions. To ensure standardized basal-view orientation, the nasal tip was aligned with the glabellar midline, and the camera axis was adjusted to achieve a frontal basal view with both nostril apertures clearly visible and symmetrically exposed. A millimetric ruler was placed at the level of the nostrils in each photograph for individual calibration. Images were analyzed using Image J software version 1.54g (National Institutes of Health, Bethesda, MD, USA). ImageJ-based analysis has been used to calculate nostril surface area ratios from basal-view photographs of patients with unilateral cleft lip and palate [7]. Each photograph was calibrated individually using the visible millimetric ruler. The nostril aperture was defined as the visible airway opening bordered laterally by the inner margins of the alar rim skin and medially by the columellar skin.

The right and left nostril apertures were manually outlined using the freehand selection tool. For each nostril, area (mm^2^), height (mm), and width (mm) were recorded separately at postoperative week 1 and postoperative month 3. A representative ImageJ-based measurement is presented in Figure 1B.

### 2.4. Observer Measurements and Reliability Analysis

All measurements were performed by two independent observers who were not involved in the surgical procedures or postoperative clinical follow-up. Although group allocation was not explicitly disclosed to the observers, a formal blinded assessment was not performed. Each observer repeated the measurements three times. For each observer, the mean of the three measurements was calculated. The final value used for statistical analysis was the average of the means from both observers.

Interobserver reliability was assessed using intraclass correlation coefficient (ICC) analysis. A two-way mixed-effects model with an absolute agreement definition was applied. As the average of the two observers’ measurements was used in the final analyses, average-measures ICC values were reported with 95% confidence intervals.

### 2.5. Symmetry Index Calculation Method

Measurements of the right and left nostrils were used to calculate symmetry indices for area, height, and width separately. The symmetry index was calculated as follows:

“Symmetry index” = “smaller value”/“larger value” × 100

Values closer to 100 indicated greater symmetry between the right and left nostrils. The change in symmetry index was calculated as

Δ“Symmetry index” = “Month-3 symmetry index” − “Week-1 symmetry index”

A positive delta value indicated an improvement in symmetry, while a negative value indicated a reduction in symmetry.

### 2.6. Statistical Analysis

Statistical analyses were conducted using the Statistical Package for the Social Sciences 30.0.0 (SPSS) (SPSS Inc., Chicago, IL, USA). Continuous variables were reported as mean ± standard deviation, and categorical variables as frequency and percentage. Normality was assessed using the Shapiro–Wilk test, histograms, and Q-Q plots.

Between-group comparisons of continuous variables were performed using Student’s *t*-test. Categorical variables were compared using the chi-square test or Fisher’s exact test. The primary analysis compared changes in the area symmetry index between the nostril retainer and control groups, while secondary analyses compared changes in the height and width symmetry indices.

Interobserver reliability was assessed using intraclass correlation coefficients for average measures with a two-way mixed-effects absolute agreement model. Subgroup correlations between baseline area asymmetry and improvement in area symmetry index were evaluated using Spearman’s correlation coefficient. A *p*-value of <0.05 was considered statistically significant.

## 3. Results

A total of 52 patients were included in the study. Twenty-six patients were assigned to the nostril retainer group and 26 patients to the control group. The mean age was 28.19 ± 6.84 (18–47) years in the nostril retainer group and 26.92 ± 7.91 (18–45) years in the control group, with no significant between-group difference (*p* = 0.539). Sex distribution was also comparable between groups, with 16 female patients in the nostril retainer group and 14 female patients in the control group (*p* = 0.575).

Surgical variability was minimized by applying a standardized primary open structural rhinoplasty protocol in both groups. Key maneuvers related to nasal tip and columellar support, including flexible tongue-in-groove technique, columellar strut grafting, spreader graft placement, and dome-binding sutures, were performed in all patients. Procedures that could directly modify nostril aperture contour, such as alar rim grafting and alar base surgery, were not performed in any patient. Cap grafting was the only variable additional tip-contouring maneuver and was performed in 20 patients in the nostril retainer group and 18 patients in the control group, with no significant between-group difference (76.9% vs. 69.2%, *p* = 0.755).

At postoperative week 1, baseline nostril symmetry indices were similar between the groups. No significant differences were observed in area symmetry index (93.16 ± 5.58 vs. 94.90 ± 4.90, *p* = 0.238), height symmetry index (95.29 ± 3.99 vs. 95.33 ± 4.93, *p* = 0.975), or width symmetry index (90.04 ± 7.87 vs. 89.84 ± 5.96, *p* = 0.917). At postoperative month 3, the area symmetry index was higher in the retainer group than in the control group, although this difference did not reach statistical significance (96.24 ± 2.72 vs. 93.90 ± 6.19, *p* = 0.083). No significant between-group differences were observed in month-3 height or width symmetry indices (*p* = 0.982 and *p* = 0.217, respectively) (Table 1).

### 3.1. Interobserver Reliability

Interobserver reliability was good to excellent for all ImageJ-based measurements, with average-measures ICC values ranging from 0.850 to 0.997 (Table S1).

### 3.2. Symmetry Changes

**Figure 2:**
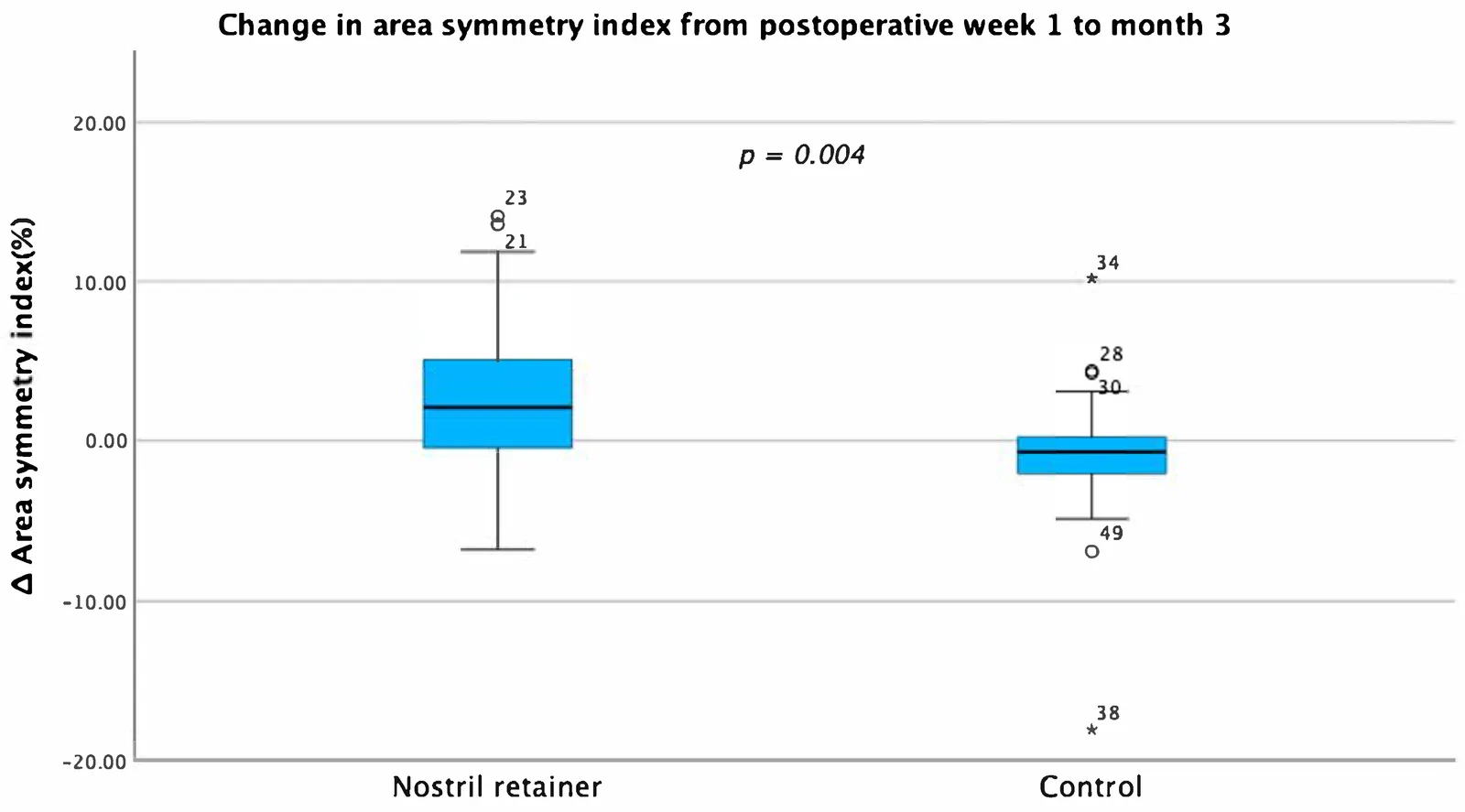
Change in area symmetry index from postoperative week 1 to month 3. The nostril retainer group showed a significantly greater improvement than the control group (*p* = 0.004).

The increase in area symmetry index from postoperative week 1 to month 3 was significantly greater in the nostril retainer group than in the control group (3.08 ± 4.99 vs. −1.00 ± 4.86; mean difference: 4.08; 95% CI: 1.34–6.82; *p* = 0.004) (Table 2). The distribution of Δ area symmetry index values by group is shown in Figure 2.

In a subgroup correlation analysis, baseline area asymmetry was strongly correlated with improvement in area symmetry index in the nostril retainer group (Spearman’s rho = 0.822, *p* < 0.001), whereas this correlation was not significant in the control group (Spearman’s rho = 0.308, *p* = 0.126).

**Figure 3:**
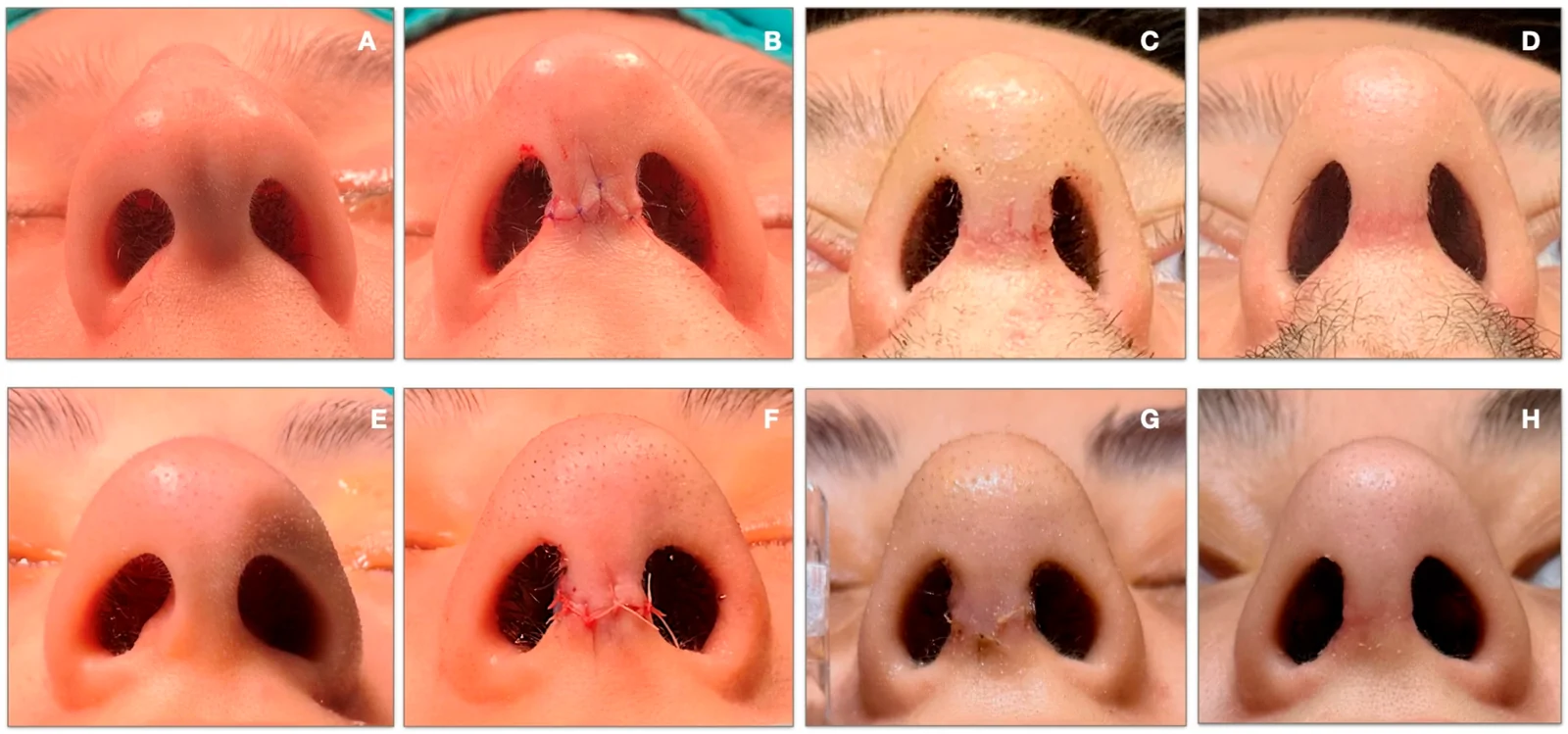
Representative serial basal-view photographs of two patients in the nostril retainer group. (**A**–**D**) First patient in the nostril retainer group: (**A**) preoperative basal view; (**B**) immediately postoperative basal view obtained in the operating room; (**C**) postoperative week-1 basal view obtained before initiation of nostril retainer use; and (**D**) postoperative month-3 follow-up basal view. (**E**–**H**) Second patient in the nostril retainer group: (**E**) preoperative basal view; (**F**) immediately postoperative basal view obtained in the operating room; (**G**) postoperative week-1 basal view obtained before initiation of nostril retainer use; and (**H**) postoperative month-3 follow-up basal view.

**Figure 4:**
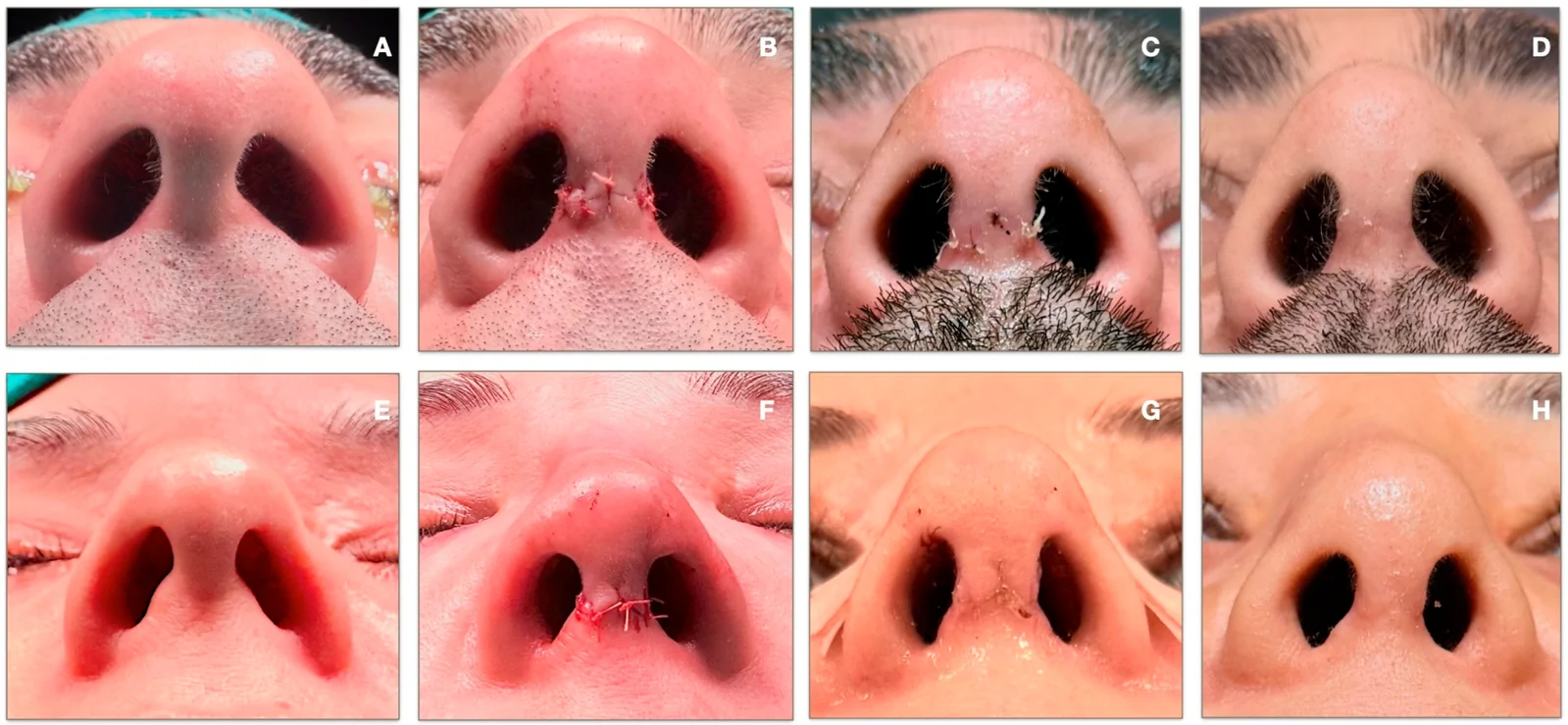
Representative serial basal-view photographs of two patients in the control group. (**A**–**D**) First patient in the control group: (**A**) preoperative basal view; (**B**) immediately postoperative basal view obtained in the operating room; (**C**) postoperative week-1 basal view; and (**D**) postoperative month-3 follow-up basal view. (**E**–**H**) Second patient in the control group: (**E**) preoperative basal view; (**F**) immediately postoperative basal view obtained in the operating room; (**G**) postoperative week-1 basal view; and (**H**) postoperative month-3 follow-up basal view.

Representative serial basal-view photographs showing the preoperative, immediately postoperative, postoperative week-1, and postoperative month-3 appearances are presented for the nostril retainer group in Figure 3 and for the control group in Figure 4.

## 4. Discussion

The principal finding of this study is that early postoperative use of nostril retainers is associated with a significant increase in nostril area symmetry following primary rhinoplasty. Between postoperative week 1 and month 3, the area symmetry index increased in the retainer group but decreased slightly in the control group (3.08 ± 4.99 vs. −1.00 ± 4.86; *p* = 0.004). In contrast, changes in height and width symmetry indices did not change significantly between groups. These results show that the benefit of nostril retainer use is primarily reflected in the overall configuration of the nostril aperture, rather than in isolated vertical or horizontal dimensions.

Nostril shape and bilateral symmetry are important components of nasal base aesthetics after rhinoplasty. Even when the dorsal profile and nasal tip contour are satisfactory, asymmetry visible on basal view may negatively influence the perception of the surgical outcome [1]. Therefore, nostril symmetry should be evaluated using both subjective aesthetic judgment and objective measurement methods. Previous studies have evaluated nostril symmetry using two-dimensional photographic measurements, including nostril width, height, area, and gap area ratios [2,8]. However, these studies were conducted in cleft populations and did not report repeated measurements by independent observers or interobserver reliability analysis. In the present study, calibrated basal-view photographs were used to directly quantify the symmetry of right and left nostril aperture area, height, and width. Two independent observers repeated measurements, and good-to-excellent ICC values supported the reproducibility of the morphometric protocol. This methodological approach may have improved measurement consistency and reduced observer-dependent variability.

The use of nostril retainers is based on the biomechanical processes of early postoperative healing. Following rhinoplasty, nostril contour is shaped by both surgical technique and factors such as edema resolution, wound healing, scar remodeling, tissue memory, and contractile forces around the alar rim and nostril aperture. In cleft rhinoplasty and nostril stenosis, postoperative splints, stents, or retainers are used to support nasal form, reduce contraction and relapse, and maintain vestibular patency [4,9]. Although reconstructive procedures differ from primary aesthetic rhinoplasty, a comparable mechanical rationale may be applicable to selected primary rhinoplasty patients. Early mechanical support could stabilize nostril tissues during the active healing phase; however, direct evidence for this mechanism in primary aesthetic rhinoplasty is limited. Therefore, the biomechanical explanation presented in this study should be regarded as a plausible hypothesis rather than a confirmed mechanism. Early nostril support may be particularly important for patients at elevated risk of postoperative nostril distortion due to substantial edema, unfavorable soft tissue characteristics or a predisposition to contracture. However, this interpretation remains hypothetical because these risk factors were not quantified individually in the current study.

The timing and duration of retainer use are critical. Pavri et al. used three-dimensional morphometric analysis to show that about two-thirds of postoperative edema resolves within the first month after rhinoplasty, though soft-tissue changes continue for several months [10]. In parallel, the wound-healing literature indicates that the early postoperative period involves fibroblast activity, collagen synthesis, extracellular matrix maturation, and wound contraction, all of which contribute to the final tissue configuration [11]. Postoperative nostril support protocols differ significantly. Some authors recommend immediate retainer fixation for 2 to 4 weeks, while others suggest delaying removable nasal stents for 2 to 3 weeks to support home use [4,12,13,14]. In the present study, retainer use began after the week-1 photographs and continued until postoperative week 5. This protocol provided early mechanical support during active edema resolution and soft-tissue remodeling, while avoiding immediate fixation and aiming to improve patient tolerability. Therefore, the improvement observed at postoperative month 3 may reflect early stabilization of the nostril aperture configuration rather than only the immediate mechanical effect of the device.

Evidence on nostril retainer use after primary rhinoplasty remains limited. Kandemir et al. studied 60 patients with primary rhinoplasty, starting retainer use on postoperative day 14 for 8 h daily over 3 months. The retainer group had significantly higher postoperative FACE-Q scores, but no significant difference in NOSE scores. Photogrammetric analysis showed lower postoperative interalar width and significant changes in certain basal view ratios [5]. Overall, their findings suggest that nostril retainer use after primary rhinoplasty may contribute to aesthetic nasal base contour and patient satisfaction. However, no significant improvement in nasal obstruction symptoms was demonstrated. The present study is consistent with this morphologic benefit, showing that retainer use was associated with improved symmetry of the nostril area; however, functional outcomes were not considered.

Findings from the cleft literature further support the use of postoperative nostril retainers. Al-Qatami et al. found smaller differences between cleft and non-cleft sides in nasal measurements, including nostril aperture width and height, among patients using postsurgical retainers [12]. Funayama et al. showed that presurgical nasal stenting combined with postsurgical retainer placement improved nasal symmetry compared to no appliance use [13]. While these studies focused on cleft populations, they support the broader principle that mechanical nostril support helps preserve nasal base and nostril form. While Rossell-Perry et al. found that postoperative nasal conformers did not significantly improve nasal symmetry in unilateral cleft lip and palate patients [15], our results suggest that nostril retainers may provide a morphometric advantage to primary rhinoplasty patients, especially those with marked early asymmetry. In the retainer group, baseline nostril-area asymmetry was strongly correlated with improvement in the area symmetry index, a relationship not observed in the control group. This finding may indicate that retainers could be more beneficial in patients with measurable nostril-area asymmetry early after surgery.

Several limitations should be acknowledged. First, this study was conducted as a prospective, non-randomized comparative observational study rather than a randomized clinical trial. The decision to use postoperative nostril retainers was based on clinical judgment rather than random allocation, introducing the potential for selection bias. Patients with more pronounced early postoperative nostril asymmetry, narrower nostril apertures, greater edema, or less favorable soft-tissue characteristics may have been more likely to receive a retainer, even though week-1 baseline symmetry indices were statistically comparable between groups. Therefore, while the results indicate an association between nostril retainer use and improved early nostril area symmetry, a definitive causal relationship cannot be established due to the absence of randomization in treatment allocation.

Second, this study focused exclusively on objective morphometric assessment of nostril aperture symmetry and did not include patient-reported outcomes or functional scores. The postoperative month 3 endpoint was selected to evaluate early nostril aperture configuration after completion of the retainer protocol and initial soft-tissue remodeling. However, longer-term follow-up would be necessary to determine the durability of these findings.

Despite these limitations, this study has several strengths. The prospective design enabled the use of a standardized photographic protocol with millimetric ruler-based calibration, supporting objective and reproducible ImageJ-based assessments. Surgical variability was reduced by including only patients operated on by a single surgeon. Furthermore, repeated measurements by two independent observers, along with good-to-excellent interobserver reliability, reinforce the consistency of the morphometric data. Importantly, this study directly evaluated the effect of nostril retainers on nostril aperture symmetry in primary aesthetic rhinoplasty, a topic that remains insufficiently explored in the existing literature.

## 5. Conclusions

Early postoperative nostril retainer use was associated with improved nostril area symmetry after primary rhinoplasty. These findings suggest that short-term mechanical support during early healing may help preserve or enhance bilateral nostril aperture configuration, particularly in terms of global area symmetry. Nostril retainers may represent a simple, noninvasive adjunct for selected patients at risk of early postoperative nostril asymmetry. Prospective randomized studies with longer follow-up and patient-reported outcomes are needed to confirm the durability and clinical relevance of this effect.

## Figures and Tables

**Table 1:** Demographic characteristics and postoperative nostril symmetry indices of the study groups.

Variable	Retainer Group, *n* = 26	Control Group, *n* = 26	*p* Value
Age, years	28.19 ± 6.84 (18–47)	26.92 ± 7.91 (18–45)	0.539 ^1^
Female, *n* (%)	16 (61.5)	14 (53.8)	0.575 ^2^
Male, *n* (%)	10 (38.5)	12 (46.2)	
Area symmetry index at week 1, %	93.16 ± 5.58	94.90 ± 4.90	0.238 ^1^
Height symmetry index at week 1, %	95.29 ± 3.99	95.33 ± 4.93	0.975 ^1^
Width symmetry index at week 1, %	90.04 ± 7.87	89.84 ± 5.96	0.917 ^1^
Area symmetry index at month 3, %	96.24 ± 2.72	93.90 ± 6.19	0.083 ^1^
Height symmetry index at month 3, %	95.45 ± 3.41	95.42 ± 4.45	0.982 ^1^
Width symmetry index at month 3, %	92.20 ± 5.20	89.98 ± 7.18	0.217 ^1^

^1^ = Student’s *t*-test; ^2^ = Chi-square test.

**Table 2:** Changes in nostril symmetry indices from postoperative week 1 to postoperative month 3.

Variable	Retainer Group	Control Group	Mean Difference	95% CI	*p* Value
Δ Area symmetry index	3.08 ± 4.99	−1.00 ± 4.86	4.08	1.34 to 6.82	0.004 ^1^
Δ Height symmetry index	0.16 ± 4.08	0.10 ± 3.37	0.06	−2.02 to 2.15	0.951 ^1^
Δ Width symmetry index	2.16 ± 7.82	0.14 ± 4.39	2.02	−1.54 to 5.58	0.257 ^2^

Values are presented as mean ± standard deviation. Δ = postoperative month 3 − postoperative week 1. ^1^: Student’s *t*-test; ^2^: Welch’s *t*-test. CI, confidence interval.

## Data Availability

The datasets generated and analyzed during the current study are not publicly available due to restrictions under the Turkish Law on the Protection of Personal Data but are available from the corresponding author upon reasonable request.

## Supplementary Materials

The following supporting information can be downloaded at: https://www.mdpi.com/article/10.3390/medicina62081436/s1, Table S1. Interobserver reliability of ImageJ-based nostril measurements.

## References

[1] Brito Í.M., Avashia Y., Rohrich R.J. (2020). Evidence-based nasal analysis for rhinoplasty: The 10-7-5 method. Plast. Reconstr. Surg. Glob. Open.

[2] Reddy S.G., Devarakonda V., Reddy R.R. (2013). Assessment of nostril symmetry after primary cleft rhinoplasty in patients with complete unilateral cleft lip and palate. J. Cranio-Maxillofac. Surg..

[3] Egan K.K., Kim D.W. (2005). A novel intranasal stent for functional rhinoplasty and nostril stenosis. Laryngoscope.

[4] Nguyen D.C., Myint J.A., Lin A.Y. (2024). The role of postoperative nasal stents in cleft rhinoplasty: A systematic review. Cleft Palate Craniofacial J..

[5] Kandemir S., Caner L.N., Bayar Muluk N., Taş B.M., Şencan Z., Cömert E. (2025). Effectiveness of postoperative nostril retainer in rhinoplasty patients. Eur. J. Plast. Surg..

[6] Erdal A.I., Genç İ.G., Pasinlioğlu B., Findikçioğlu K. (2021). Flexible Tongue-in-Groove Technique and Its Effectiveness Compared to Classic Tongue-in-Groove and Columellar Strut Graft Techniques: A Retrospective Analysis of 237 Open Rhinoplasty Cases. Plast. Reconstr. Surg..

[7] Sulaiman F.K., Haryanto I.G., Hak S., Nakamura N., Sasaguri M., Ohishi M. (2013). Fifteen-year follow-up results of presurgical orthopedics followed by primary correction for unilateral cleft lip nose in program SEHATI in Indonesia. Cleft Palate-Craniofacial J..

[8] Uchino N., Hoshi K., Takato T., Okayasu M., Saijo H., Nakatsuka T., Ohkubo K. (2021). Follow-up study of nasal deformity in cleft lip and palate patients: Morphological changes and image analysis with growth. Oral Sci. Int..

[9] Zhang S., Wu M., Chen J., Yin J., Sakran K.A., Wang Y., Zeng N., Yang C., Shi B., Huang H. (2023). The necessity of nostril retention application after secondary unilateral cleft rhinoplasty. Laryngoscope.

[10] Pavri S., Zhu V.Z., Steinbacher D.M. (2016). Postoperative edema resolution following rhinoplasty: A three-dimensional morphometric assessment. Plast. Reconstr. Surg..

[11] Sarian M.N., Zulkefli N., Zain S., Maniam S., Fakurazi S. (2023). A review with updated perspectives on in vitro and in vivo wound healing models. Turk. J. Biol..

[12] Al-Qatami F., Avinoam S.P., Cutting C.B., Grayson B.H., Shetye P.R. (2022). Efficacy of postsurgical nostril retainer in patients with unilateral cleft lip and palate treated with presurgical nasoalveolar molding and primary cheiloplasty–rhinoplasty. Plast. Reconstr. Surg..

[13] Funayama E., Yamamoto Y., Oyama A., Furukawa H., Murao N., Hayashi T., Maeda T., Osawa M. (2019). Comparison of nasal symmetry between presurgical nasal stenting and postsurgical nasal retainer placement in unilateral clefts. J. Craniofacial Surg..

[14] Bezuhly M. (2014). Rapid intraoperative fabrication of an inexpensive, reliable nasal stent for use after primary cleft nasal repair. Cleft Palate Craniofacial J..

[15] Rossell-Perry P., Romero-Narvaez C., Gavino-Gutierrez A., Figallo-Hudtwalcker O. (2023). Postoperative nasal conformers in cleft rhinoplasty: Are they efficacious?. J. Craniofacial Surg..

