# Supplementary material for: The Effect of Nostril Retainer Use on Nostril Aperture Symmetry After Primary Aesthetic Rhinoplasty
Source: Medicina (Kaunas). 2026 Jul 24;62(8):1436. doi: 10.3390/medicina62081436 (PMC13514553; doi:10.3390/medicina62081436)
Supplement: Supplementary file 1 [file medicina-62-01436-s001.zip › medicina-4407292-supplementary.pdf]

Table S1. Interobserver reliability of ImageJ-based nostril measurements

| Measurement | Average-measures ICC | 95% CI | p value |
|---|---|---|---|
| Week 1 right nostril area | 0.997 | 0.994–0.998 | <0.001 |
| Week 1 right nostril height | 0.989 | 0.981–0.994 | <0.001 |
| Week 1 right nostril width | 0.988 | 0.979–0.993 | <0.001 |
| Week 1 left nostril area | 0.997 | 0.994–0.998 | <0.001 |
| Week 1 left nostril height | 0.984 | 0.973–0.991 | <0.001 |
| Week 1 left nostril width | 0.993 | 0.987–0.996 | <0.001 |
| Month 3 right nostril area | 0.975 | 0.956–0.986 | <0.001 |
| Month 3 right nostril height | 0.950 | 0.913–0.971 | <0.001 |
| Month 3 right nostril width | 0.992 | 0.986–0.996 | <0.001 |
| Month 3 left nostril area | 0.959 | 0.928–0.976 | <0.001 |
| Month 3 left nostril height | 0.938 | 0.892–0.964 | <0.001 |
| Month 3 left nostril width | 0.850 | 0.739–0.913 | <0.001 |

ICC, intraclass correlation coefficient; CI, confidence interval.
